# circ_WASF2 regulates ferroptosis by miR-634/ GPX4 signaling in pancreatic cancer

**DOI:** 10.1007/s12672-024-01001-4

**Published:** 2024-05-05

**Authors:** Tao Liu, Xing-ming Xie, Ya-peng He, Jia-yao Zhang, Jun-ying Mou

**Affiliations:** 1 Department of Hepatobiliary Surgery, The Central Hospital of Enshi Tujia and Miao Autonomous Prefecture, No. 158, Wuyang Avenue, Enshi, 445000 Hubei China; 2 Department of Hepatobiliary Surgery, The Key Laboratory of Advanced Interdisciplinary Studies Center, The First Affiliated Hospital of Guangzhou Medical University, Guangzhou, 510120 Guangdong China; 3 Department of Anesthesiology, The Central Hospital of Enshi Tujia and Miao Autonomous Prefecture, No. 158, Wuyang Avenue, Enshi, 445000 Hubei China

**Keywords:** circ_WASF2/miR-634/GPX4, Ferroptosis, Lipid peroxidation, Reactive oxygen species (ROS), Pancreatic cancer

## Abstract

**Purpose:**

Pancreatic cancer (PC) is one of the most lethal malignant gastrointestinal tumors (GI) characterized by a poor prognosis. Ferroptosis is an emerging programmed cell death that plays an essential role in the progression of various cancers. Ferroptosis is driven by iron-dependent phospholipid peroxidation and is regulated by mitochondrial activity, lipid peroxidation, and reactive oxygen species (ROS). The function and mechanism of ferroptosis in PC need more research.

**Methods:**

The levels of circRNAs, miRNAs, and mRNAs were detected by quantitative real-time polymerase chain reaction (qRT-PCR). Western blot was used for protein detection. CCK8 assays were used to detect cell proliferation. Cell death, lipid peroxidation, ROS, and Fe^2+^ were detected by indicted kits. Dual-luciferase reporter and RNA pull-down assays were conducted to confirm the interaction between circRNAs, miRNAs, and mRNAs.

**Results:**

In this research, we found that circular RNA hsa_circ_0000003(circ_WASF2) was upregulated in pancreatic cancer cells. The silence of circ_WASF2 inhibited cancer proliferation and increased cell death by increasing ferroptosis accompanied by up-regulation of lipid peroxidation, ROS, and Fe^2+^. Further studies showed that circ_WASF2 could attenuate ferroptosis by targeting miR-634 and the downstream glutathione peroxidase 4 (GPX4). GPX4 has been well-reported as a central factor in ferroptosis. Our research revealed a new pathway for regulating ferroptosis in PC.

**Conclusion:**

In summary, we have determined that circ_WASF2/miR-634/GPX4 contributed to ferroptosis-induced cell death, and provided a possible therapeutic target in PC.

**Supplementary Information:**

The online version contains supplementary material available at 10.1007/s12672-024-01001-4.

## Introduction

Pancreatic cancer is one of the most lethal malignancies, ranking as the fourth leading cause of cancer-related mortality [1]. Pancreatic ductal adenocarcinoma (PDAC) comprises more than 95% of PC [2]. PC still remains a deadly malignancy with the development of detection techniques and management. The 5-year survival rate of PC patients is only about 9% [3, 4]. The main factors that lead to the poor prognosis of PC are the difficulty of early detection, rapid progression, and chemoresistance [5, 6]. The main treatments for PC consist of surgery, target therapy, chemotherapy, and radiotherapy [7]. Standard therapy for resectable PC is surgery followed by adjuvant chemotherapy. However, most patients diagnosed with advanced PC have lost the opportunity for surgery due to the lack of appropriate early detection and screening methods [8].

Recently, ferroptosis has been reported to be more and more important in breast cancer, live cancer, gastric cancer, and pancreatic cancer [9–12]. Ferroptosis is a newly found form of iron-dependent non-apoptotic regulatory programmed cell death that is induced by reactive oxygen species (ROS) and lipid peroxidation [13]. Glutathione peroxidase 4 (GPX4) is a glutathione peroxidase that has been reported to be a key factor in the ferroptosis process [14]. GPX4 plays a critical role in the conversion of lipid hydroperoxides into lipid alcohols. GPX4 malfunction causes the accumulation of lipid peroxides which will induce ferroptosis. Ferroptosis is reported to be a tumor suppressor, and targeting GPX4 might be a promising target for therapy [15]. Many researches have reported that the free iron concentration in PC is higher than in normal cells, and ferroptosis can lead to PC cell death [16, 17]. Targeting ferroptosis in tumor cells might be a promising therapeutic method for PC.

Interestingly, several researchers have found that noncoding RNAs also play essential roles in regulating ferroptosis. Circular RNAs (circRNAs) are newly found noncoding RNAs with a covalently closed loop. Circular RNAs are tissue specificity and abundance conservation. Circular RNAs play various regulatory functions in downstream target genes, packaging of the genome, imprinting of genomic, and chromatin modification. CircRNAs are reported to serve as essential regulatory factors in cancer progression [18]. Recently, the function of circRNAs in ferroptosis has been discovered in various cancers. CirIL4R could facilitate tumorigenesis and inhibit ferroptosis in hepatocellular carcinoma by regulating the miR‐541‐3p/GPX4 axis [18]. Circular RNA circ_0067934 attenuates ferroptosis of thyroid cancer cells by miR-545-3p/SLC7A11 signaling [19]. Circ ASAP2 decreased inflammation and ferroptosis in diabetic nephropathy through SOX2/SLC7A11 by miR‑770‑5p [19].

Our research focused on the function of circRNAs in PC.hsa_circ_0000003(circ_WASF2) has 329 bp in length and is located in chr1:27747415-27747744. In the present research, we identified a novel function of circ_WASF2 in attenuating ferroptosis and tumor growth by regulating miR-634/ GPX4 in PC.

## Materials and methods

### Cell culture and transfections

Two human pancreatic cancer cell lines PANC-1 and BXPC-3 were purchased from the American Type Culture Collection (ATCC, Manassas, VA, USA). BxPC-3 was maintained in RPMI 1640 medium supplemented with 1 mM sodium pyruvate, 2.5 g/L glucose, and 10% fetal bovine serum (FBS). PANC-1 cells were cultured in Dulbecco’s modified Eagle medium (DMEM) supplemented with 2 mM l-glutamine and 10% FBS. MicroRNA mimic, inhibitor, and corresponding control oligo nucleotides (NC) were obtained from General Biosystem Company (Anhui, China). Small interfering RNA targeting circ_WASF2 (si-circ_WASF2) and circ_WASF2 overexpression vector were designed and synthesized by RiboBio Biotechnology (Guangzhou, China). Transfections of all microRNA oligo nucleotides and vectors were performed using Lipofectamine 3000 (Invitrogen).

### RNase R treatment assay

Total RNA was extracted and incubated for 20 min at 37 °C with 3U/μg of RNase R (Geneseed, Guangzhou, China). 2 μg RNA was used for RNase R treatment assay. qRT-PCR was used to detect the expression of circ_WASF2. GAPDH was used as an internal reference.

### Actinomycin D(ActD)treatment

2X10^5^ cells were seeded into a 6-well plate and cultured overnight. Cells were harvested at 0, 4,8,12, and 24 h with 2 mg/L ActD treatment (Sigma, USA). The RNAs were extracted and analyzed by qRT-PCR.

### RNA extraction and quantitative real-time PCR (qRT PCR) analysis

TRIzol Reagent (Thermo Fisher Scientific, Waltham, MA, USA) was used to isolate RNA from PC cells. The PrimeScript RT Reagent Kit (Takara, Dalian, China) was used for reverse transcription. MicroRNA levels were detected using the StepOnePlus TM Real-Time PCR System (Thermo Fisher Scientific, MA, US). Relative expression levels of circRNA, mRNA and miRNA were normalized to GAPDH and U6, respectively. The analysis was performed using the 2^−△△CT^ method.

### Western blot analysis

RIPA buffer (Solarbio, Beijing, China) with proteinase inhibitors was used to isolate proteins from PC cells. The BCA kit (Beyotime) was used to determine the concentration of proteins. 20 μg protein were used for detection. SDS–polyacrylamide gels were applied to separate different proteins. The proteins were then transferred to polyvinylidene difluoride (PVDF) membranes. The PVDF membranes were cultured with 5% fat-free milk for 2 h at 37 °C. The membranes were then cultured with primary antibodies overnight at 4 °C. Anti-GPX4 (ab125066, 1:2000), anti-beta-catenin (ab32572, 1:1000) were obtained from Abcam (Cambridge, UK). The membranes were washed with PBST for 5 min three times and incubated with the indicated secondary antibodies for 45 min. Proteins were detected using an Odyssey infrared imaging instrument (LI-COR, USA).

### RNA pull-down assay

Biotin-labeled probes targeting circ_WASF2 and a control probe (a random oligo probe) were incubated with streptavidin beads (Invitrogen, USA) at 25 °C for 3 h. Incubated PC cell lysis with beads at 4 °C overnight. Subsequently, the beads were washed three times and the beads were eluted. Then, the level was detected by qRT-PCR.

### Dual-luciferase reporter assay

The wild type (WT) or mutant sequence of circ_WASF2 was cloned into pGL3 plasmids (Genechem, Shanghai, China). The wild-type (WT) or mutant sequence of GPX4 3′UTR was cloned into the pGL3 vector. The pGL3 plasmids and miR-634 were co-transfected into PC cells using Lipofectamine 3000. The relative activities of firefly Luciferase and Renilla Luciferase were measured and analyzed using a luciferase reporter assay system (Promega, Madison, WI, USA).

### CCK8 assay

5000 PC cells were seeded in 96-well plates per well and cultured overnight. 10 μl CCK-8 (Dojindo, Japan) was added per well and stored at 37 °C for 2 h. The absorbance of 450 nm was detected by a microplate reader (Tecan Trading AG, Switzerland).

### Cell death assay

Cell death was measured with the Cell Death Detection ELISA*plus* kit (Roche, Germany) following the manufacturer's protocol.

### Measurement of iron, lipid peroxidation, and reactive oxygen species levels

Fe^2+^ levels were measured using an iron assay kit (Sigma, USA, MAK025). The levels of reactive oxygen species (ROS) were measured using a Cellular ROS Assay Kit (Abcam, United States). The lipid peroxidation (MDA) levels were measured by a lipid peroxidation (MDA) assay kit (Abcam, United States).

### Statistical analysis

Data are shown as the mean ± SD. Student’s t-test was used to detect statistical differences between the two groups. One-way analysis of variance (ANOVA) was used to detect the statistical difference between multiple groups. P < 0.05 (two-tailed) was considered.

## Results

### circ_ WASF2 was upregulated in PC with high stability

**Fig. 1:**
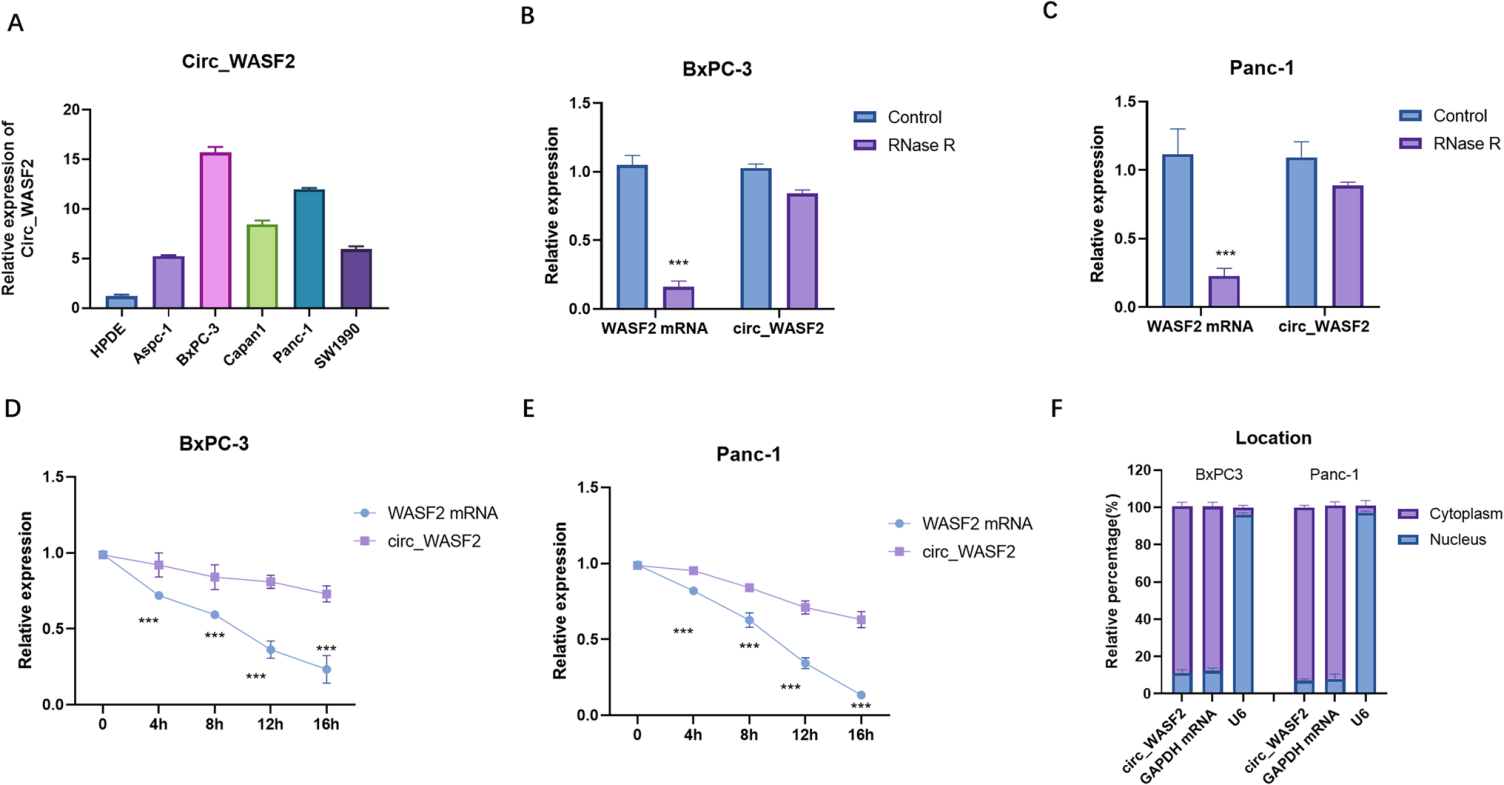
circ_ WASF2 was upregulated in PC with high stability. **A** qRT-PCR detection of circ_ WASF2 was performed in PC cell lines and normal pancreatic ductal cells HPDE. **B** qRT-PCR detection of circ_ WASF2 and WASF2 mRNA with specially designed divergent and convergent primers with RNase R treatment was performed in BxPC3. **C** qRT-PCR detection of circ_ WASF2 and WASF2 mRNA with specially designed divergent and convergent primers with RNase R treatment in Panc-1. **D** qRT-PCR detection of circ_ WASF2 in BxPC3 with ActD treatment. **E** qRT-PCR detection of circ_ WASF2 in Panc-1 with ActD treatment. **F** qRT-PCR was performed after RNA nucleocytoplasmic separation, with GAPDH, U6 as markers of the cytoplasm and nucleus in BxPC3 (left) and Panc-1 (right)

circ_WASF2 levels in PC cells (Aspc-1, BxPC-3, Capan-1, Panc-1 and SW1990) were higher than normal pancreatic ductal cells HPDE (Fig. 1A). The stability of circ_WASF2 was detected by the RNase R and Actinomycin D assays. The RNase R digestion experiment showed that circ_WASF2 was more resistant to RNA exonuclease than its linear mRNA in both BxPC-3 (Fig. 1B) and Panc-1 (Fig. 1C). Actinomycin D assays showed that circ_WASF2 had a longer half-life than linear WASF2 mRNA in both BxPC-3 (Fig. 1D) and Panc-1 (Fig. 1E). The nucleus-cytoplasmic separation assay showed that circ_WASF2 was predominantly localized in the cytoplasm of PC cells (Fig. 1F). Taken together, these results showed that circ_WASF2 was highly expressed in PC and was mainly located in the cytoplasm.

### Silence of circ_ WASF2 induced cell death of PC through increasing ferroptosis

**Fig. 2:**
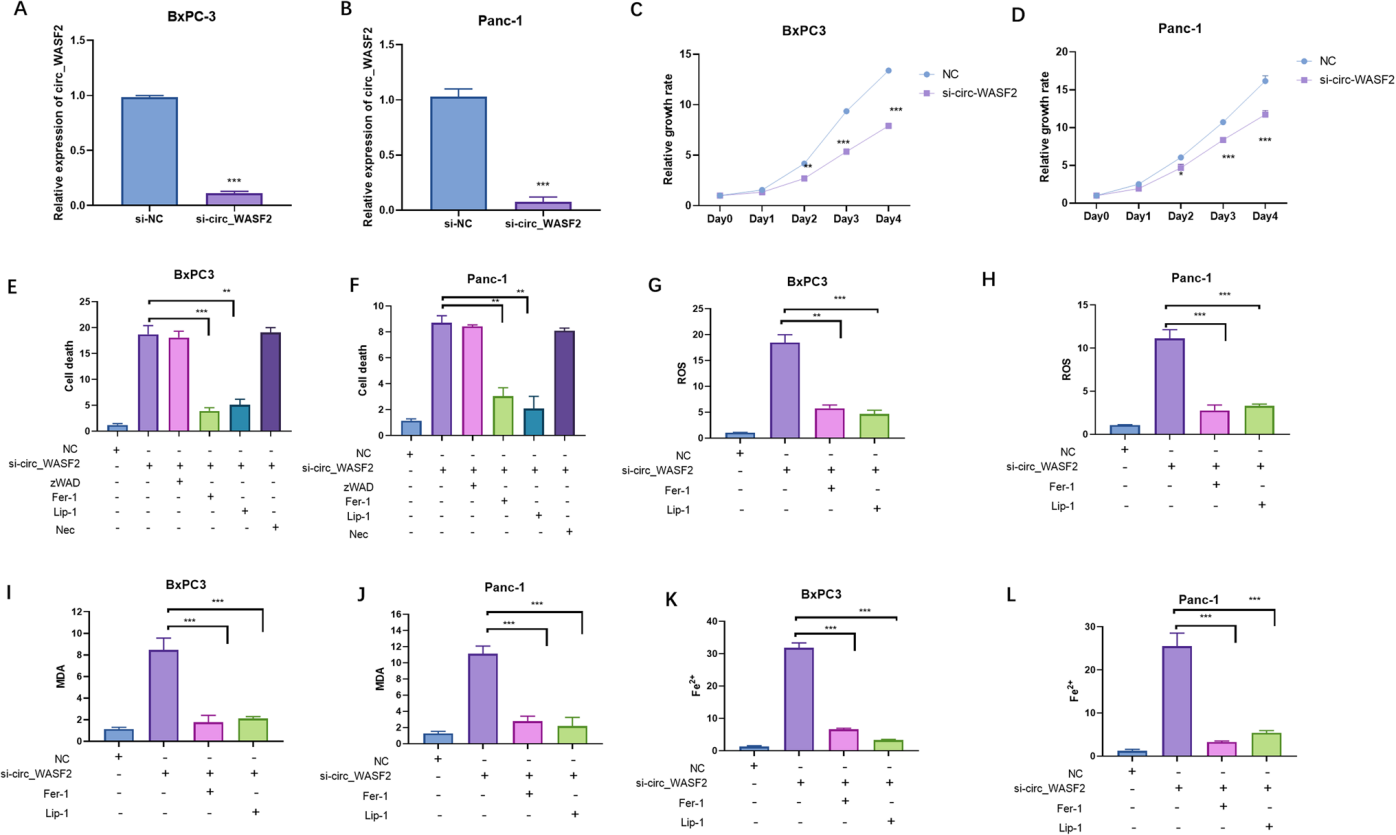
The silence of circ_ WASF2 inhibited PC proliferation through increasing ferroptosis. **A**, **B** qRT-PCR assays were used to detect the expressions of circ_WASF2 in both BxPC3 and Panc-1.nc is short for nonsense control. **C**, **D** CCK8 assays were used to detect BxPC3 and Panc-1 proliferation in both BxPC3 and Panc-1. **E**, **F** Cell deaths were detected in BxPC3 and Panc-1 cells that were transfected with indicated siRNAs and treated with different inhibitors. **G**, **H** BxPC3 and Panc-1 cells were transfected with the indicated siRNAs and treated with or without ferroptosis inhibitors (Fer-1, Lip-1) for 24 h, and ROS levels were detected. **I**, **J** MDA levels were detected in BxPC3 and Panc-1 with indicated treatment. **K**, **L** Fe^2+^ levels were detected in BxPC3 and Panc-1 with indicated treatment

This research investigated the function of circ_ WASF2 in PC. In PC cells, it was found that the silence of circ_ WASF2 inhibited proliferation and enhanced cell death compared to the control group in both BxPC3 (Fig. 2A, C, E) and Panc-1 (Fig. 2B, D, F). To determine the type of cell death induced by circ_ WASF2, apoptosis inhibitor (z.VAD.FMK, 10 mM), ferroptosis inhibitors (Fer-1 10 mM, Lip-1 20 mM) and necrosis inhibitor (Necrosulfonamide, NEC) were applied in pancreatic cancer. The results showed that ferroptosis inhibitors could significantly attenuate the cell death induced by silence of circ_ WASF2 both in BxPC3 (Fig. 2E) and Panc-1 (Fig. 2F). Also, apoptosis and necrosis inhibitors could not influence circ_WASF2-induced cell death in PC cells (Fig. 2E, F). To further confirm whether the cell death induced by circ_WASF2 was triggered by ferroptosis, experiments were performed to detect the changes in ROS, MDA, and Fe2^+^ with the silence of circ_WASF2. The results showed that silence of circ_WASF2 could increase the levels of ROS, MDA, and Fe2^+^, and the increase could be reversed by Fer-1 and Lip-1(Fig. 2G–L). These results showed that the silence of circ_WASF2 increased the cell death of PC cells through ferroptosis.

### circ_WASF2 regulates the expression of miR-634

**Fig. 3:**
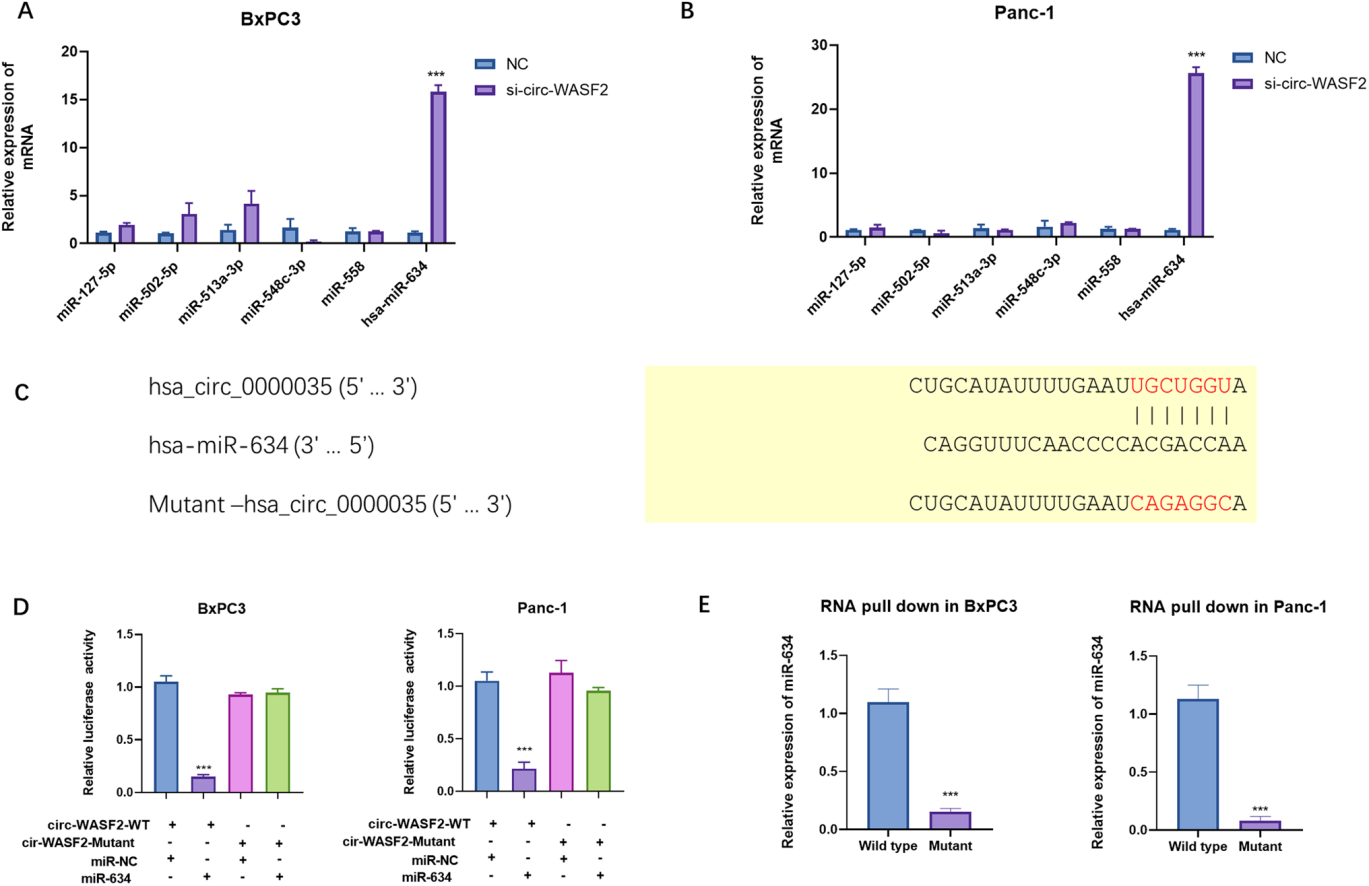
circ_WASF2 acted as a sponge for miR-634. **A**, **B** qRT-PCR assays were performed in BxPC3 and Panc-1 with silence of circ_WASF2. **C** Mutation of binding sites of circ_WASF2. nc is short for nonsense control. **D** Luciferase activity assays were performed with transfection of pGL3-circ_WASF2 or pGL3-mutant -circWASF2 in BxPC3 and Panc-1. **E** RNA pull-down assays were performed both in BxPC3 and Panc-1 cells

CircRNAs have been well-reported to act as sponges to regulate the expressions of microRNAs [20]. The sub-cellular fractionation experiment in Fig. 1 showed that circ_WASF2 mainly existed in the cytoplasm of PC cells suggesting that circ_WASF2 could act as sponges for microRNA. Then, bioinformatical tools (circInteractome) predicted that 6 possible microRNAs could bind to circ_WASF2 (supplementary Fig. 1). qRT-PCR was used to detect the predicted microRNA levels in both BxPC3 and Panc-1 with the silence of circ_WASF2 (Fig. 3A, B). The results showed that the silence of circ_WASF2 could significantly up-regulate the expression of miR-634 (Fig. 3A, B). To investigate the influence of circ_WASF2 on miR-634, we constructed wild-type and mutant circ_WASF2 pGL3 plasmids. Luciferase activity assay showed that miR-634 could significantly decrease the luciferase activity of wild-type circ_WASF2. And, the mutation of the binding sites in circ_WASF2 could significantly block the influence of miR-634 on circ_WASF2 (Fig. 3C, D). RNA pull-down assay showed that wild-type circ_WASF2 had direct interaction with miR-634, and the mutation abrogated the bindings between circ_WASF2 and miR-634 (Fig. 3E). These results confirmed that circ_WASF2 could regulate the expression of miR-634.

### MiR-634 targets GPX4, which regulates ferroptosis

**Fig. 4:**
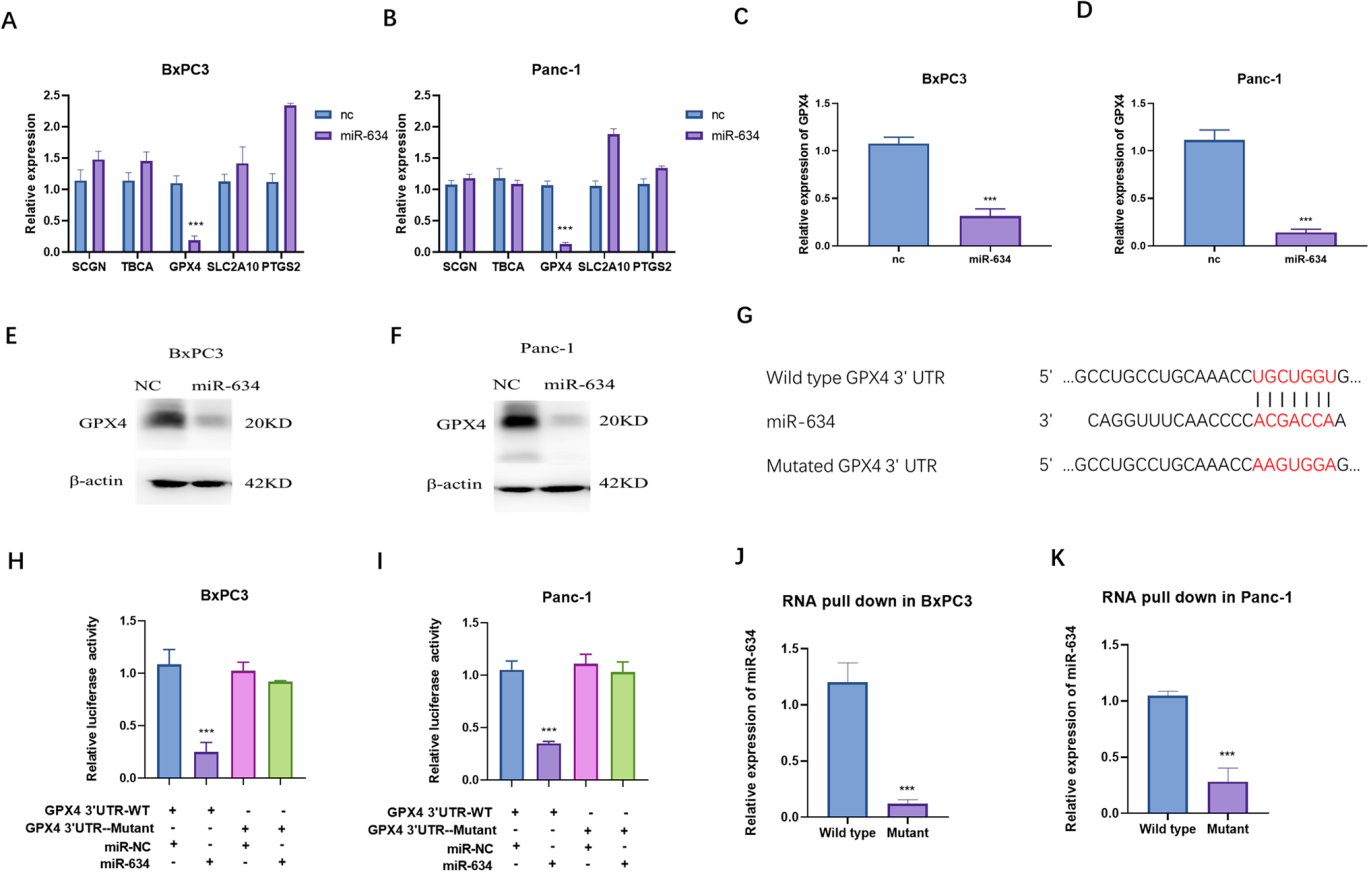
MiR-634 targeted GPX4. **A**, **B**. qRT-PCR assays were used to detect the expressions of miR-634 predicted genes in BxPC3 and Panc-1. nc is short for nonsense control. **C**–**F** The mRNA and protein levels of GPX4 were confirmed by qRT-PCR and western blot in BxPC3 and Panc-1. **G** Mutation of the binding site on GPX4 3’UTR. **H**, **I** Luciferase activity assays were performed with transfections of indicated plasmids in BxPC3 and Panc-1. **J**, **K** RNA pull-down assays were performed in BxPC3 and Panc-1

In this study, we attempt to identify the potential targets of miR-634. qRT-PCR assays were used to detect possible targets which were predicted by Targetscan. The results showed that miR-634 transfection negatively regulated the expression of GPX4 mRNA both in BxPC3 (Fig. 4A) and Panc-1 (Fig. 4B). We detect the influence of miR-634 on GPX4 mRNA and protein levels. The results showed that miR-634 transfection could negatively regulate both GPX4 mRNA and protein levels in both BxPC3 (Fig. 4C, E) and Panc-1 (Fig. 4D, F). To confirm the influence of miR-634 on GPX4, we constructed mutated plasmids of binding sites (Fig. 4G). The results of the luciferase activity assay showed that miR-634 could negatively regulate the luciferase activity of wild-type GPX4 3'UTR and could not influence the luciferase activity of mutated GPX4 3’UTR (Fig. 4H, I). The RNA pull-down assay showed that miR-634 has an interaction with GPX4 3’UTR, and mutation of the binding sites could abrogate the interaction between miR-634 and GPX4 3’UTR (Fig. 4J, K). These results confirmed that miR-634 could regulate GPX4 expression.

### circ_WASF2/miR-634/GPX4 regulated cell death through ferroptosis

**Fig. 5:**
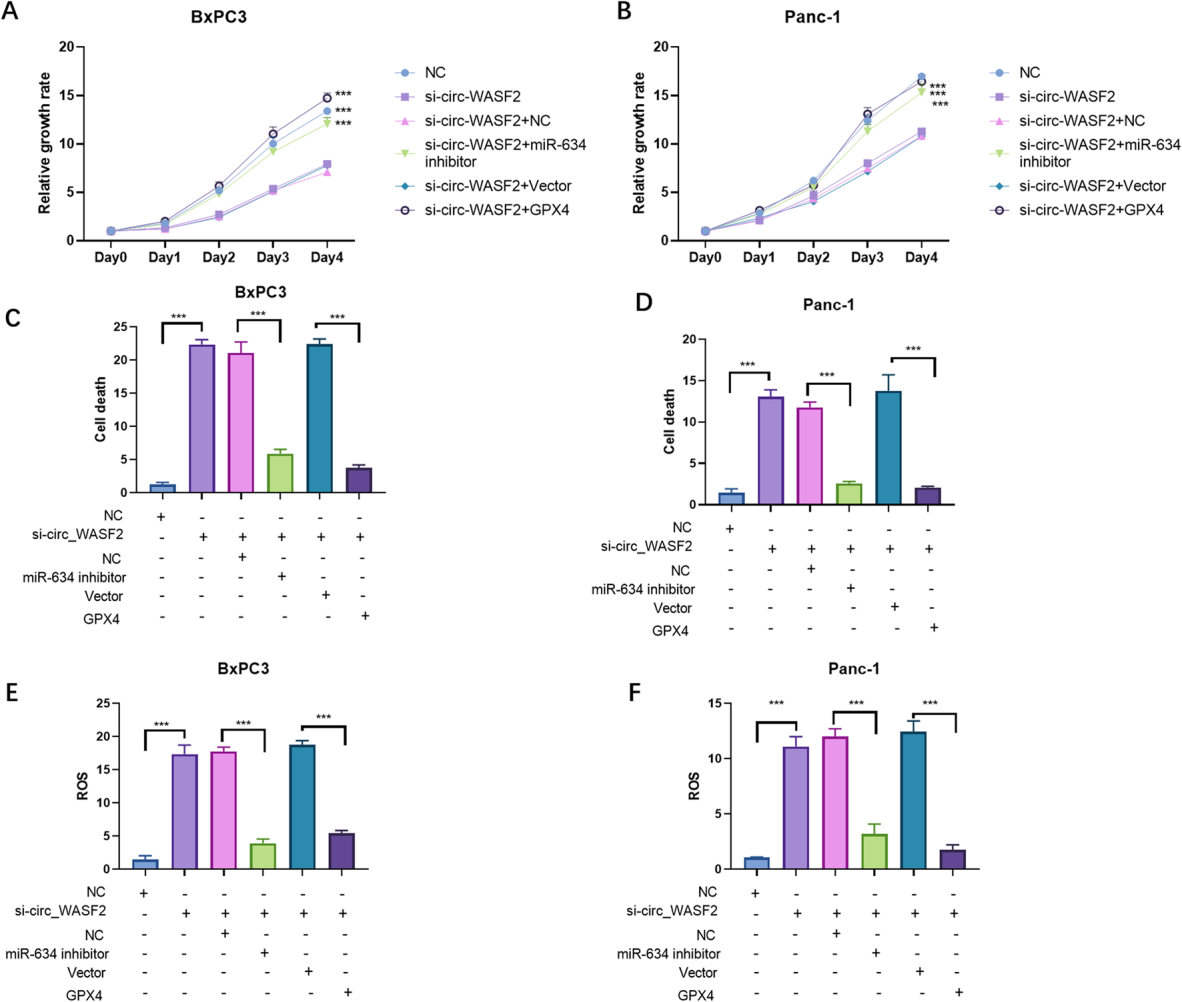
circ_WASF2/miR-634/GPX4 regulated PC cell death through ferroptosis. **A**, **B**. CCK8 assays were performed to detect cell growth in BxPC3 and Panc-1 with the indicated transfections. NC is short for nonsense control. **C**, **D**. Cell death assays were performed to detect the cell growth in BxPC3 and Panc-1 with the indicated transfections. **E**, **F**. ROS assays were performed to detect cell growth in BxPC3 and Panc-1 with the indicated transfections

Rescue experiments were carried out to detect the effect of the circ_WASF2/miR-634/GPX4 on the growth rate and cell death of PC cells. The CCK8 assay showed that miR-634 inhibitor and GPX4 re-expression reversed the circ_WASF2-silence induced proliferation inhibition in both BxPC3 (Fig. 5A) and Panc-1 (Fig. 5B). Cell death assays were also performed in BxPC3 and Panc-1 cells with the miR-634 inhibitor and GPX4 re-expression. The results showed that the miR-634 inhibitor and GPX4 re-expression reversed cell death increase induced by circ_WASF2 silence in both BxPC3 (Fig. 5C) and Panc-1 (Fig. 5D). To further confirm whether circ_WASF2/miR-634/GPX4 regulated cell death through ferroptosis, we performed rescue experiments to detect ROS. The ROS results showed that the miR-634 inhibitor and GPX4 re-expression reversed the circ_WASF2-silence induced ROS increasement (Fig. 5E, F). Together, these results suggest that circ_WASF2/miR-634/GPX4 regulates proliferation and cell death through ferroptosis in PC.

## Discussion

Recently, increasing numbers of research have reported that circRNAs play an important role in the progression of various cancers, including prostate cancer [21], ovarian cancer (OC) [22], osteosarcoma (OS) [23], and hepatocellular carcinoma (HCC) [24]. In our research, the results showed that circ_WASF2 acts as an important factor in ferroptosis-related cell death. circ_WASF2 is more highly expressed in PC cells than in normal pancreatic ductal cells HPDE. The silence of circ_WASF2 inhibited cell growth and increased cell death in PC. Further experiments revealed that silence of circ_WASF2 inhibited cancer cell proliferation and increased cell death by increasing ferroptosis accompanied by up-regulation of lipid peroxidation (MDA), reactive oxygen species (ROS), and Fe^2+^. Through biological information analysis and biological experiments, we found that circ_WASF2 exerts its function in ferroptosis mainly through the miR-634/GPX4 pathway.

CircRNAs have been a rising star in noncoding RNA biological research. More and more circRNAs have been found to play an important role in various disease progressions with the development of high-throughput sequencing technology and bioinformatics analysis. One of the main functions of circRNAs is acting as sponges to promote the progression of cancer by antagonizing the expression and function of miRNA. We predicted the potential downstream targets of circ_WASF2 through bioinformatics tools (CircInteractome, Targetscan) and initially identified six candidate targets. qRT-PCR, RNA pulldown, and luciferase activity assays were performed to confirm the targets. The results showed that miR-634 was the target of circ_WASF2.

microRNAs are the critical downstream regulators of circRNA-mediated cancer progression. MiR-634 has been reported to exhibit antitumor activities toward hepatocellular carcinoma via Rab1A and DHX33 [25]. miR-634 inhibits human vascular smooth muscle cell proliferation and migration in hypertension through the Wnt4/β-catenin pathway [26]. miR-634 decreases the radio resistance of human breast cancer cells by targeting STAT3. MiR-634 sensitizes glioma cells to temozolomide by targeting CYR61 through the Raf-ERK signaling pathway [27]. miR-634 restores drug sensitivity in resistant ovarian cancer cells by targeting the Ras-MAPK pathway [28]. MiR-634 has been reported to act as a tumor suppressor in various cancer progressions. In our study, miR-634 inhibited proliferation and increased ferroptosis-induced cell death in PC by targeting GPX4. We found that circ_WASF2 acted as the upstream regulator of miR-634, providing more information for miR-634-induced tumor suppression.

Recent research has shown that ferroptosis plays an important role in the progression of cancers, especially PC. Ferroptosis is a kind of non-apoptotic regulatory programmed cell death that is iron-dependent and is caused by lipid peroxidation and plasma membrane rupture. Numerous studies have shown that ferroptosis can lead to the development of gastrointestinal (GI) tumors. The concentration of Fe^2+^ in PC is higher than that in normal cells. Research on ferroptosis in PC is of great value for PC therapy.

Importantly, glutathione peroxidase 4 (GPX4) has been well-reported as a central factor in ferroptosis.GPX4 is a selenocysteine-containing protein with strong antioxidant activities. The expression of GPX4 could be regulated at various levels, including transcription, translation, post-translational, and epigenetic modifications. It might be a promising therapy for cancer by targeting GPX4 and inducing ferroptosis. Previous research has uncovered that circRNAs could regulate ferroptosis through various pathways. circRNAs could actively mediate the process of ferroptosis. CircRNA circSTIL inhibits ferroptosis in colorectal cancer via miR‐431/SLC7A11 axis [29].CircRNA1615 inhibits ferroptosis via modulation of autophagy by the miRNA152-3p/LRP6 Axis in Cardiomyocytes of Myocardial Infarction [30].Circular RNA circBLNK promotes osteosarcoma progression and inhibits ferroptosis in osteosarcoma cells by sponging miR-188-3p and regulating GPX4 expression [11]. The function of circRNAs in regulating ferroptosis has gained more and more attention.

In our investigation, we found that circ_WASF2 was up-regulated in PC cells. The silence of circ_WASF2 inhibited cancer cell proliferation and increased cell death by increasing ferroptosis accompanied by up-regulation of lipid peroxidation, reactive oxygen species (ROS), and Fe^2+^. Further studies showed that circ_WASF2 could regulate GPX4 expression through miR-634. Our research uncovered a new pathway to regulate ferroptosis in PC.

In future work, we will continue to investigate the function of circ_WASF2 in pancreatic cancer. We will investigate the upstream regulation of circ_WASF2 expression in pancreatic cancer. We also want to perform more experiments to confirm the circ_WASF2/miR-634/GPX4 pathway in clinical samples. What’s more, we will investigate our findings in other cancers. We hope our research can be confirmed in other cancers.

## Conclusion

In summary, we have determined that circ_WASF2/miR-634/GPX4 contributed to ferroptosis-induced cell death and provided a possible therapeutic application in PC. The results showed a new relationship between circRNAs and ferroptosis in pancreatic cancer and provided new targets for ferroptosis-induced cell death. Our researcher uncovered the complex interaction between circRNAs and ferroptosis which provides new thoughts about programmed cell death.

### Supplementary Information

Additional file 1 (DOCX 639 KB)

## Data Availability

The datasets used are available on reasonable request. Further enquiries can be directed to the corresponding author.
