# Supplementary material for: circ_WASF2 regulates ferroptosis by miR-634/ GPX4 signaling in pancreatic cancer
Source: Discov Oncol. 2024 May 5;15:143. doi: 10.1007/s12672-024-01001-4 (PMC11070409; doi:10.1007/s12672-024-01001-4)
Supplement: Supplementary file 1 — Additional file 1 (DOCX 639 KB) [file 12672_2024_1001_MOESM1_ESM.docx]

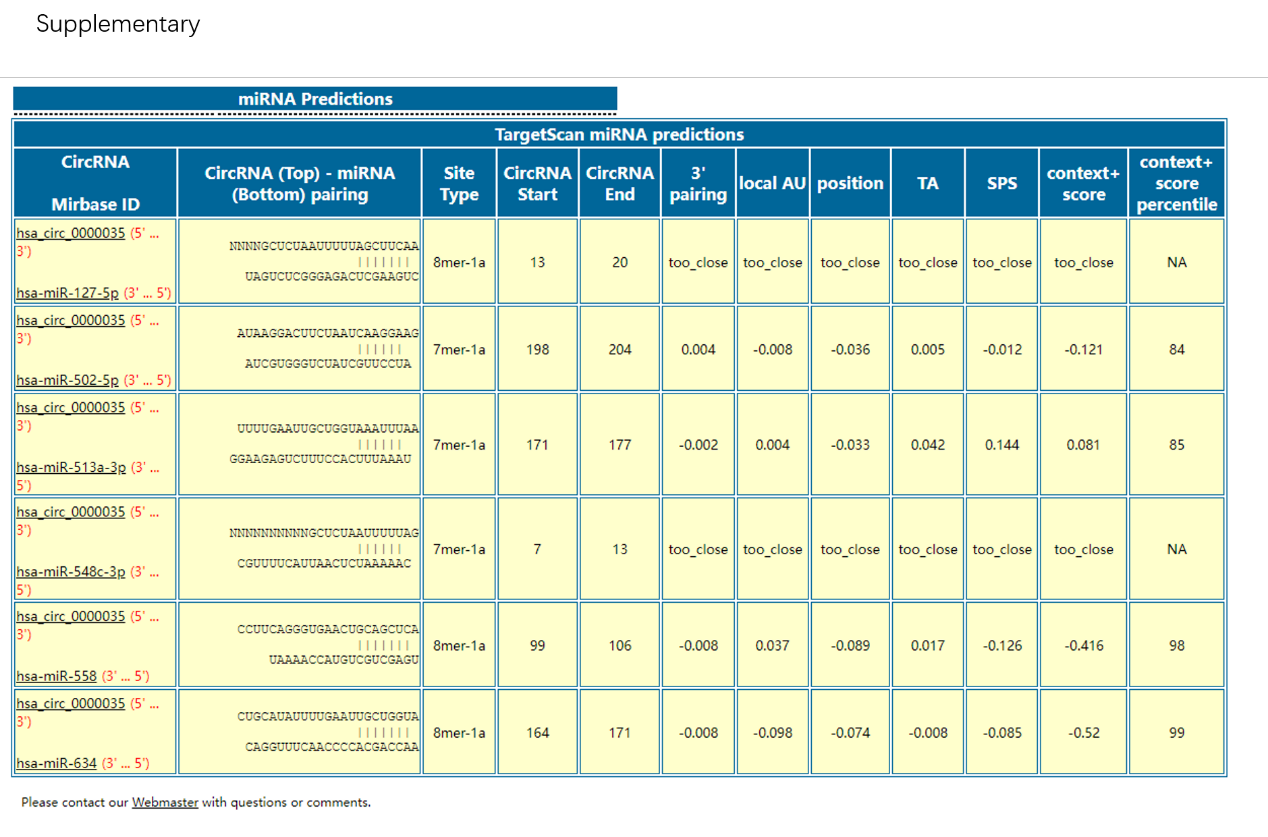


Supplementary Figure 1.

circInteractome predicted that 6 possible microRNAs could bind to circ_WASF2.
